# Multidimensional Evaluation of Guideline-Based Generative Artificial Intelligence Responses in Traditional Chinese: A Ménière’s Disease Study

**DOI:** 10.3390/life16101649

**Published:** 2026-09-30

**Authors:** Mien-Jen Lin, Yun-Chiao Wen, Li-Chun Hsieh, Chin-Kuo Chen

**Affiliations:** 1 Department of Medical Education, Chang Gung Memorial Hospital, Taoyuan 333423, Taiwan; j565243782@gmail.com; 2 Department of Otolaryngology-Head and Neck Surgery, Linkou Chang Gung Memorial Hospital, Taoyuan 333423, Taiwan; joanna14631@gmail.com; 3 Department of Otolaryngology-Head and Neck Surgery, Mackay Memorial Hospital, Taipei 104217, Taiwan; lichunhsieh1978@gmail.com; 4 Department of Audiology and Speech Language Pathology, Mackay Medical University, New Taipei 252005, Taiwan; 5 Department of Medicine, Mackay Medical University, New Taipei 252005, Taiwan; 6 Department of Otolaryngology-Head and Neck Surgery, Keelung Chang Gung Memorial Hospital, Keelung 204201, Taiwan; 7 School of Medicine, College of Medicine, Chang Gung University, Taoyuan 333323, Taiwan

**Keywords:** generative artificial intelligence, Ménière’s disease, clinical practice guidelines, Chinese language models, concordance analysis

## Abstract

Background: The reliability of generative artificial intelligence for Chinese medical information remains uncertain. This study evaluates the concordance and linguistic performance of three generative artificial intelligence systems in generating Chinese information on Ménière’s disease against clinical practice guidelines. Methods: Seventeen questions, adapted from the Key Action Statements of the American Academy of Otolaryngology–Head and Neck Surgery guidelines, were posed to ChatGPT o4-mini-high, Gemini 2.5 Pro, and Grok 3 (51 total responses). Responses were assessed for guideline concordance, communication features, and readability with matched analyses (Cochran’s Q and Friedman tests), with Holm–Bonferroni correction across nine communication characteristics. Results: Correctness rates did not differ significantly among the three models (ChatGPT o4-mini-high: 100%, Gemini 2.5 Pro: 100%, Grok 3: 94.1%; Q = 2.00, *p* = 0.368). Six of the nine communication characteristics differed significantly, with moderate to large effect sizes (Kendall’s W = 0.26–0.76), including guideline quotation, citation quality, key point emphasis and recommendations beyond the guideline. The proportion of difficult words also differed significantly (*p* = 0.0033). Gemini 2.5 Pro had a lower proportion of difficult words than ChatGPT o4-mini-high (adjusted *p* = 0.040) and Grok 3 (adjusted *p* = 0.0002). Conclusion: High guideline concordance does not necessarily indicate reliable citations, effective communication, or accessible language. These findings reflect responses generated under single-query conditions rather than consistent model performance, highlighting the need for expert oversight in clinical use.

## 1. Introduction

### 1.1. Background and Motivation

Ménière’s disease (MD) is a chronic inner-ear disorder characterized by recurrent vertigo, fluctuating sensorineural hearing loss, tinnitus and aural fullness [1]. Its variable clinical course requires evidence-based recommendations covering diagnosis, treatment, rehabilitation and patient counselling. The American Academy of Otolaryngology–Head and Neck Surgery (AAO-HNS) clinical practice guideline (CPG) provides 17 Key Action Statements (KASs) that offer an explicit reference standard for these aspects of care [2].

Generative artificial intelligence (GenAI) has advanced rapidly with improvements in machine learning, computational capacity, and data storage, enabling the generation of fluent text and images across domains, including medicine [3,4,5,6,7,8,9]. Its growing use as a source of health information is particularly relevant in non-English-speaking settings. In Chinese-speaking regions, clinicians often rely on English-language guidelines when equivalent local guidance is unavailable, while patients seeking information independently may encounter unverified sources. This creates a gap in access to evidence-based medical information that is linguistically and culturally appropriate. GenAI could help bridge this gap through translation, summarization, and explanation; however, its reliability in specialized medical domains, particularly in languages other than English, remains uncertain [10,11,12]. In addition, GenAI and Large language models (LLMs) are two distinct but related concepts. LLMs, a specific type of GenAI, are designed primarily to understand and generate language output, and it is this function that the present study evaluates [9,13]. Throughout this article, we use “GenAI” when referring to the systems as products, reserving “LLMs” for the underlying language-generation capability.

### 1.2. Overview of the Proposal

We applied a structured, three-dimensional evaluation approach to GenAI-generated medical text in Traditional Chinese. KASs from the published CPG were converted into Chinese questions that preserve their wording, intent, and polarity, then submitted once to each system under standardized conditions. Responses were evaluated for (i) concordance with guideline recommendations, (ii) predefined clinical content and communication characteristics, and (iii) reading difficulty using a Chinese-specific text analyzer. Responses were matched by question and compared using paired non-parametric tests. We applied this protocol to the AAO-HNS MD guideline using three GenAI models: ChatGPT o4-mini-high, Gemini 2.5 Pro, and Grok 3.

### 1.3. Contributions and Assumptions

This study makes three principal contributions. First, it provides a head-to-head evaluation of three widely used GenAI systems against an established MD guideline using Traditional Chinese prompts. Second, it extends guideline-based evaluation beyond concordance by separately examining source verifiability, clinical content and communication characteristics, and Chinese lexical accessibility. Third, it demonstrates how these complementary dimensions can identify differences that would be obscured by accuracy-based evaluation alone. Interpretation of the study depends on two assumptions. The translated questions were intended to preserve the clinical intent and polarity of the original KASs; the American guideline was treated as the reference standard despite possible differences in local clinical practice. Overall, this is a medical evaluation study that applies a structured, multidimensional approach to assess guideline-based GenAI responses in Traditional Chinese. MD is the clinical test case.

### 1.4. The Structure of the Paper

The remainder of the paper is structured as follows: Section 2 reviews related research. Section 3 states the problem and the research questions. Section 4 defines the concepts, instruments and statistical constructs on which the proposed method depends. Section 5 describes the materials and methods in detail for reproduction. Section 6 specifies the evaluation design and experimental testing procedure. Section 7 reports the results together with their analysis. Section 8 discusses the findings, the domain of applicability of the proposal, and open research questions. Section 9 offers closing observations.

## 2. Related Research

Before conducting our research, we performed a narrative review on PubMed and Google Scholar to search articles with similar topics published from 2022 to 2025, using keywords such as GenAI, LLM, guideline, concordance and comparison. In these research works, GenAI was tested with examinations of different specialties, such as an audiologist qualification exam and ophthalmology residency exams [14,15,16]. Also, responses produced by GenAI were benchmarked against established guidelines [17,18,19,20,21,22,23,24]. These studies evaluated GenAI’s ability to recall factual information and apply it to specific questions, as well as solving problems with its logical reasoning [8,25]. Most of these studies primarily aimed to provide supporting evidence of using GenAI as an accurate and reliable tool for assisting with clinical decision-making, patient education and healthcare professional education [9,14,26,27]. However, limited works have addressed the textual characteristics among different models [11,12,27]. In addition, most of the responses were evaluated by reviewers or directly compared to standard answers.

Furthermore, a study investigated the performance of ChatGPT models when answering frequently asked questions in the AAO-HNS published materials on MD, focusing on accuracy, reliability and readability [18]. However, this study was designed with English-language questions based on patient education materials, assessed with English-based readability metrics and principally on one platform (ChatGPT). Some other studies suggested that GenAI’s performance may be language-dependent [10,11,12,19,24]. Specifically, there is currently no study focusing on multiple GenAI models’ performance in Chinese against established guidelines on MD.

## 3. Problem Statement

Existing evaluation studies often use guideline concordance as the primary indicator of response quality [17,21,22,24]. However, a guideline-concordant response may still contain unverifiable references, unsupported additional recommendations, ineffective communication, or linguistically difficult content. These dimensions therefore require separate assessment. The present study evaluates guideline-derived Traditional Chinese responses across three domains: guideline concordance, clinical content and communication characteristics, and reading difficulty.

This study investigated the performance of three GenAI systems—ChatGPT o4-mini-high, Gemini 2.5 Pro, and Grok 3—when answering Chinese prompts aligned with the AAO-HNS CPG for MD. In this study, we attempted to answer the following questions:

(1) What is each model’s concordance with the guidelines?
(2) How do the models differ in terms of clinical content and communication features?
(3) What is the readability of the Chinese outputs?
(4) Are there statistically significant inter-model differences in guideline concordance, textual features, or lexical accessibility metrics?

## 4. Preliminaries

### 4.1. Guideline Reference Standard

The 2020 AAO-HNS CPG for MD contains 17 KASs covering diagnosis, assessment, treatment, rehabilitation, patient education, and outcome evaluation [2]. Each statement provides a specific recommendation and supporting text based on the available evidence, such as recommending an intervention, recommending against it, or presenting it as an option. In this study, each KAS and its supporting text served as the reference standard for evaluating whether a generated response accurately conveyed the corresponding guideline recommendation.

### 4.2. Evaluation Dimensions and Chinese Lexical Accessibility

Generated responses were evaluated across three complementary dimensions: guideline concordance, clinical content and communication characteristics, and lexical accessibility. Common English readability measures, such as the Flesch Reading Ease Score and Flesch–Kincaid Grade Level, are not directly applicable to Chinese due to fundamental linguistic differences, including the absence of inter-word spacing and an equivalent concept of syllables per word [18,27,28]. We therefore used the Chinese Readability Index Explorer (CRIE) 3.0 linguistic analyzer to quantify measurable characteristics of the generated Chinese text, including character count, word count, difficult word count, sentence count, average words per sentence, and the proportion of difficult words [11,29]. A difficult word was defined according to the graded vocabulary database used by CRIE [29]. As the CRIE built-in readability score for Chinese medical text was not available for this study, these measures were seen as indicators of lexical accessibility rather than as a validated reading level or measure of patient comprehension.

## 5. Materials and Methods

**Figure 1:**
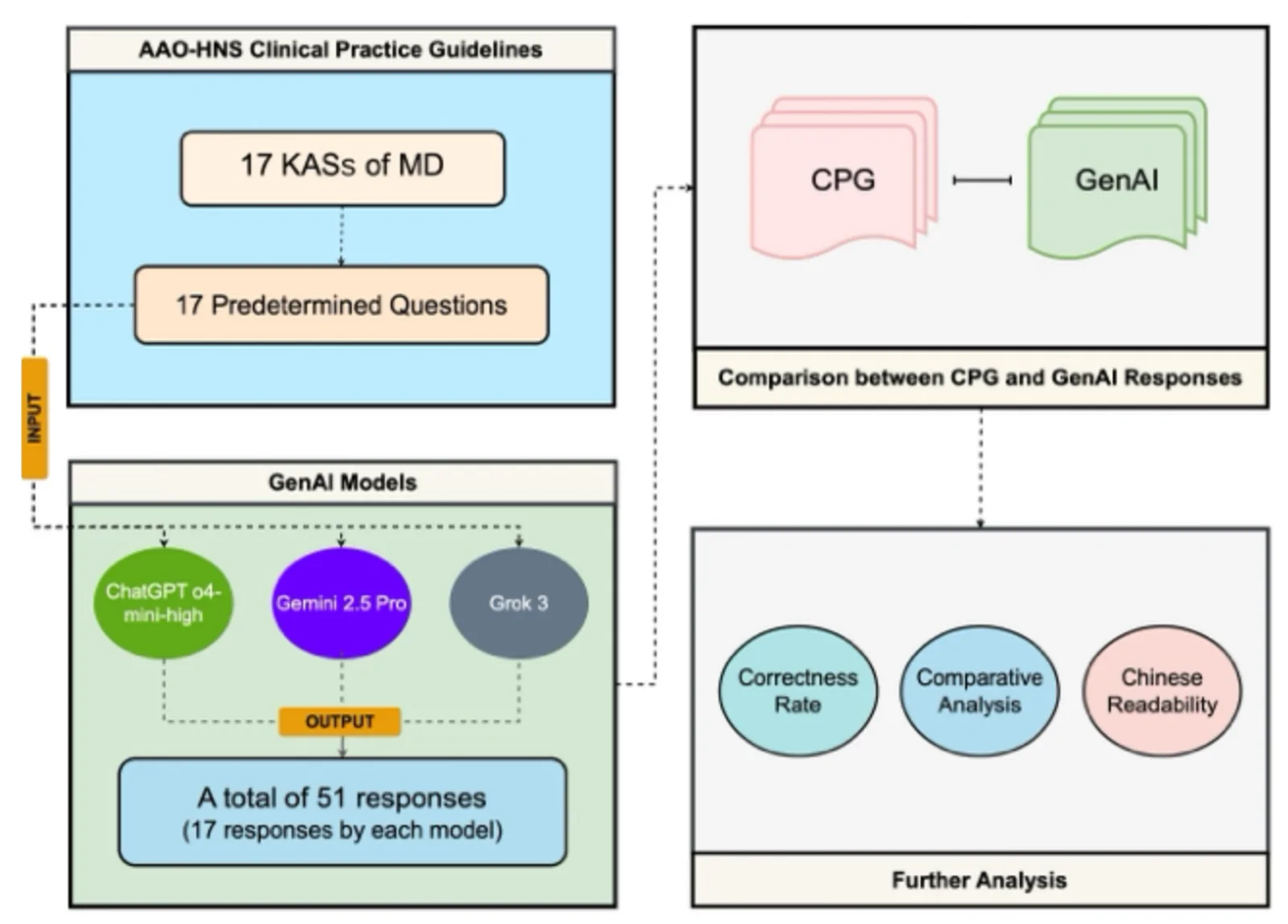
Study workflow: Seventeen questions were designed based on the seventeen KASs for MD in the AAO-HNS CPG and submitted to three GenAI systems, yielding a total of 51 responses (17 from each model). The responses were then compared with the CPG and further analyzed for correctness, clinical and communicative characteristics, and Chinese readability.

The study protocol was reviewed by the Institutional Review Board (IRB) of the Chang Gung Medical Foundation and was determined to be exempt from full review (approval no. 202500464B1; 31 March 2025). No human participants or patient data were involved. The study workflow is shown in Figure 1.

### 5.1. Study Design

This cross-sectional, question-matched study compared the responses of three GenAI systems to 17 identical Traditional Chinese questions derived from the KASs of the AAO-HNS CPG for MD. Each system generated 17 responses, yielding 51 responses for evaluation of guideline concordance, clinical content and communication characteristics, and lexical accessibility.

### 5.2. Construction of the Question Set

One Traditional Chinese question was developed from each of the 17 KASs. Each question preserved the wording, clinical intent, and polarity of the corresponding KAS without adding clinical information or altering the strength or direction of the recommendation. The questions were designed to assess the systems’ ability to reproduce established guideline recommendations rather than perform multi-step clinical reasoning or case-based decision-making. English translations of the 17 questions are presented in Table 1, while the original Chinese prompts and complete system responses are provided in Supplement S1.

### 5.3. System Selection and Interface Configuration

Three widely available GenAI systems were evaluated using models accessible under the authors’ subscription tiers: ChatGPT o4-mini-high (ChatGPT Plus), Gemini 2.5 Pro (free tier), and Grok 3 (free tier). These were the most capable models available to the authors on each platform during data collection. All questions were submitted through the standard consumer web interfaces under fixed conditions. A new chat session was started for each question, and web search was manually enabled before each query. No custom instructions, stored memory, personalization, file attachments, system prompts, role instructions, or formatting instructions were used. All other settings remained at platform defaults. Also, as web search was enabled, the evaluated outputs represent the performance of each deployed consumer system, including its retrieval functionality.

### 5.4. Data Collection

All responses were collected on 12 April 2025. Each question was submitted once to each system in Traditional Chinese, and the first complete response was retained verbatim without follow-up prompting, clarification, or regeneration. Single-query sampling was used to characterize the first response generated under the specified conditions; response consistency across repeated queries was not assessed.

### 5.5. Response Assessment

#### 5.5.1. Guideline Concordance

Responses were assessed against the corresponding KAS and its supporting text and classified as correct, partially correct, incorrect, or non-answer, based on the method of a previous study [22]:

(1) Correct: Responses that fully align with the guidelines regarding both patient approaches and therapeutic management.
(2) Partially Correct: Responses that are generally consistent with the guidelines but lacking important details.
(3) Incorrect: Responses that conflict with or deviate from the established guidelines.
(4) Non-answer: Responses contain irrelevant content or just restate the question.

Two board-certified otolaryngologists independently evaluated all 51 responses after system identifiers were removed and the responses were randomized. Both raters completed their assessments independently before consensus discussion. Pre-consensus inter-rater agreement was evaluated. Disagreements were subsequently resolved by consensus, and the final consensus ratings were used for analysis.

#### 5.5.2. Clinical Content and Communication Characteristics

Nine clinical content and communication characteristics were predefined for response evaluation (Table 2). Extensiveness, presence of misleading information, and quality of reference were adapted from a previous study, while consulting professionals and quoting the KAS were adapted from related studies [17,18,21,22]. Key point emphasis and plain-language analogies were informed by the Centers for Disease Control and Prevention Clear Communication Index (CDC-CCI) and Patient Education Materials Assessment Tool (PEMAT), respectively [30,31]. Recommendations to physicians and doctor–patient communication were defined by the authors to capture additional characteristics not covered by these existing measures. Each characteristic was assessed as present (“Yes”) or absent (“No”) according to predefined criteria. Detailed operational definitions and representative examples are provided in Supplement S2. For quality of reference, cited sources were evaluated for bibliographic verifiability and accuracy, source credibility, and consistency with the claims they supported. A response met the criterion only when all cited sources satisfied these requirements. Responses without identifiable citations were classified as not meeting the quality-of-reference criterion because source verifiability could not be established. For presence of misleading information, the entire response was reviewed, including clinical claims extending beyond the corresponding KAS. A response was rated “Yes” if at least one clinical claim contradicted the guideline or could not be verified against the supporting medical literature.

#### 5.5.3. Lexical Measures

Each response was analyzed using the CRIE 3.0 linguistic analyzer. Extracted measures included total character count, total word count, difficult word count, sentence count, and mean words per sentence. For each response, the proportion of difficult words was calculated by dividing the difficult word count by the total word count. Responses were analyzed individually, with aggregate values calculated across the 17 responses from each system.

## 6. Evaluation and Testing

### 6.1. Outcomes and Experimental Design

Three sets of responses were evaluated: ChatGPT o4-mini-high, Gemini 2.5 Pro, and Grok 3. As the same 17 questions were submitted to all three systems, responses were matched by question, which served as the unit of analysis (*n* = 17 matched question sets). Three outcome domains were evaluated: guideline concordance, clinical content and communication characteristics, and lexical measures. Guideline concordance and the nine clinical content and communication characteristics were recorded as binary outcomes for each response. Lexical measures were assessed at the individual-response level. For each of the 51 responses, the proportion of difficult words and total word count were analyzed separately as a measure of lexical difficulty. Individual words, characters, and sentences were not treated as independent observations.

### 6.2. Data Analysis

All statistical analyses were performed in Python (version 3.11) using SciPy and statsmodels. All tests were two-sided, with statistical significance set at *p* < 0.05. Differences among the three systems in guideline concordance and the nine clinical content and communication characteristics were assessed using Cochran’s Q test. Pairwise comparisons were performed for all outcomes using exact McNemar tests and are reported for completeness; comparisons for outcomes without a significant overall difference were considered descriptive. Kendall’s W was reported as the effect size for the overall comparison, while discordant pairs were reported for pairwise comparisons. For lexical measures, responses were matched by question and did not meet normality assumptions. Differences among the three systems were therefore assessed using the Friedman test, followed by pairwise Wilcoxon signed-rank tests. Descriptive statistics are presented as medians with interquartile ranges (IQRs) and means with standard deviations (SDs).

### 6.3. Multiple-Comparison Adjustment

To account for multiple testing, the Holm–Bonferroni procedure was applied across the nine clinical content and communication characteristics, which were treated as one family of outcomes. Guideline concordance and lexical measures were treated as separate outcome families. For outcomes requiring pairwise comparisons, the three pairwise tests were adjusted as a separate family using the same procedure. Both unadjusted and Holm-adjusted *p*-values are reported where applicable.

### 6.4. Inter-Rater Agreement

Pre-consensus inter-rater agreement for guideline concordance was assessed using percentage agreement, Cohen’s κ, and prevalence-adjusted bias-adjusted kappa (PABAK) [33]. Cohen’s κ was reported as the conventional chance-corrected measure of agreement, while PABAK was additionally reported because the predominance of correct responses resulted in substantial category imbalance [34]. Rater-level classifications before consensus are provided in Supplement S3.

## 7. Results

### 7.1. Guideline Concordance

**Figure 2:**
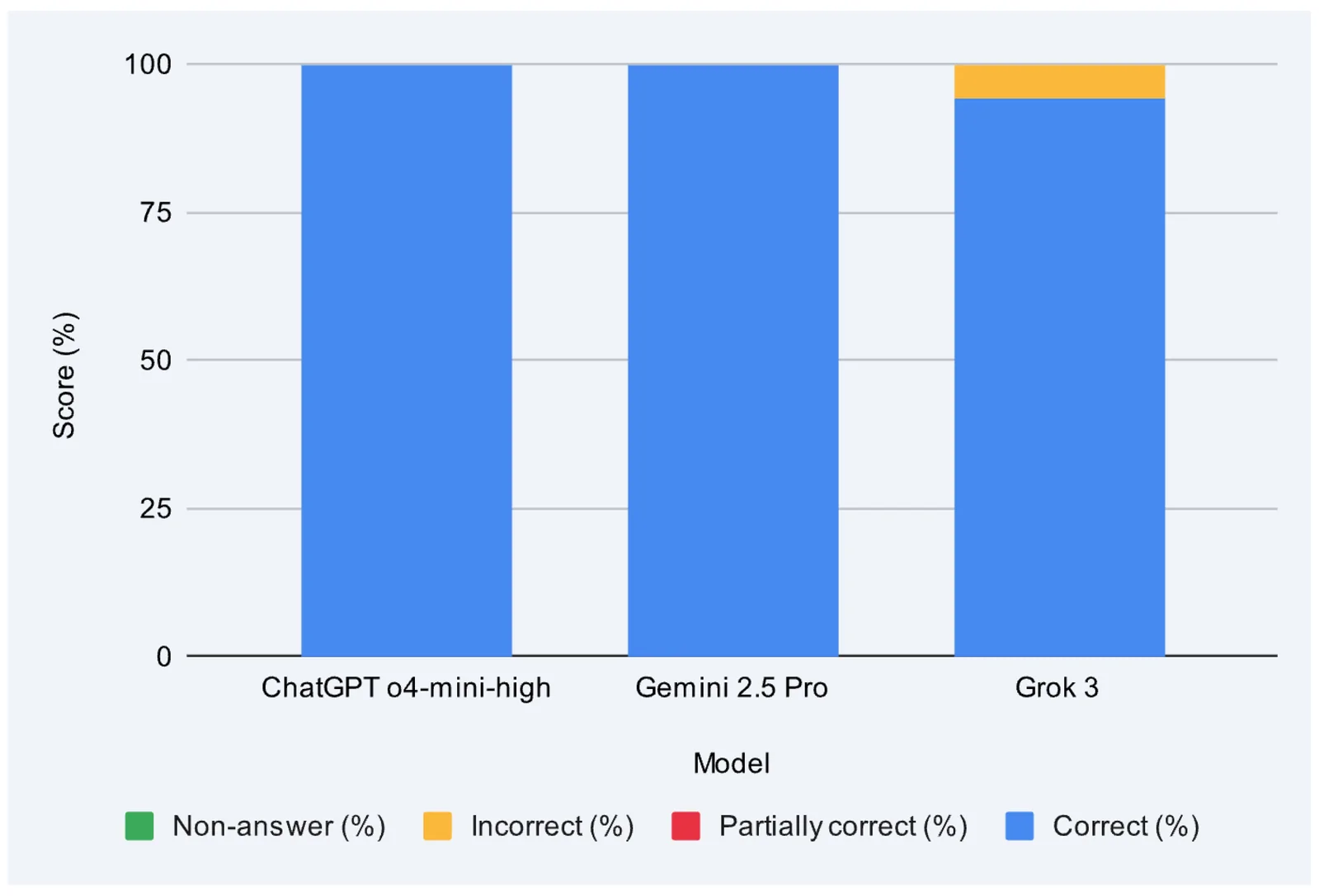
Comparison of correctness rates of ChatGPT o4-mini-high, Gemini 2.5 Pro, and Grok 3.

Of the 17 questions, ChatGPT o4-mini-high and Gemini 2.5 Pro answered all correctly (100%), while Grok 3 answered 16 correctly (94.1%). No partially correct or non-answer responses were observed. There was no significant difference in guideline concordance among the three models (Cochran’s Q = 2.00, *p* = 0.368, Kendall’s W = 0.06). Pairwise comparisons also showed no significant differences (all Holm-adjusted exact McNemar *p* = 1.000). The only incorrect response was generated by Grok 3, which suggested that positive pressure therapy, such as the Meniett device, could be considered after failure of conservative treatment (Table 3). This contradicted KAS 10, which recommends against positive pressure therapy based on evidence showing no clinical benefit [2]. Before consensus, the two raters agreed on 50 of 51 responses (98.0%), with the only disagreement involving this Grok 3 response. Cohen’s κ was 0 and PABAK was 0.96. Detailed rater assessments are provided in Supplement S3, and the comparison of correctness rates is shown in Figure 2.

### 7.2. Clinical Content and Communication Features

Significant differences among the models’ responses were identified in six of the nine evaluated characteristics after Holm adjustment: quoting KAS (Q = 26.00, adjusted *p* < 0.0001, W = 0.76), recommendations to physicians (Q = 18.43, adjusted *p* = 0.0008, W = 0.54), key point emphasis (Q = 16.00, adjusted *p* = 0.0023, W = 0.47), quality of reference (Q = 12.67, adjusted *p* = 0.0107, W = 0.37), extensiveness (Q = 9.33, adjusted *p* = 0.0470, W = 0.27), and doctor–patient communication (Q = 8.86, adjusted *p* = 0.0477, W = 0.26). No significant differences were found for plain-language analogies, misleading information, or consulting professionals (all adjusted *p* > 0.05). For pairwise comparisons, ChatGPT o4-mini-high quoted the KAS significantly more often than both Gemini 2.5 Pro and Grok 3 (both adjusted *p* = 0.0007), and provided higher-quality references than Grok 3 (adjusted *p* = 0.0059). Recommendations to physicians were more frequent in Grok 3 (adjusted *p* = 0.0007) and Gemini 2.5 Pro (adjusted *p* = 0.0156) than in ChatGPT o4-mini-high. Key point emphasis was observed only in Gemini 2.5 Pro, which differed significantly from both other models (both adjusted *p* = 0.0234). Complete pairwise comparisons for all clinical content and communication characteristics are provided in Supplement S4.

**Figure 3:**
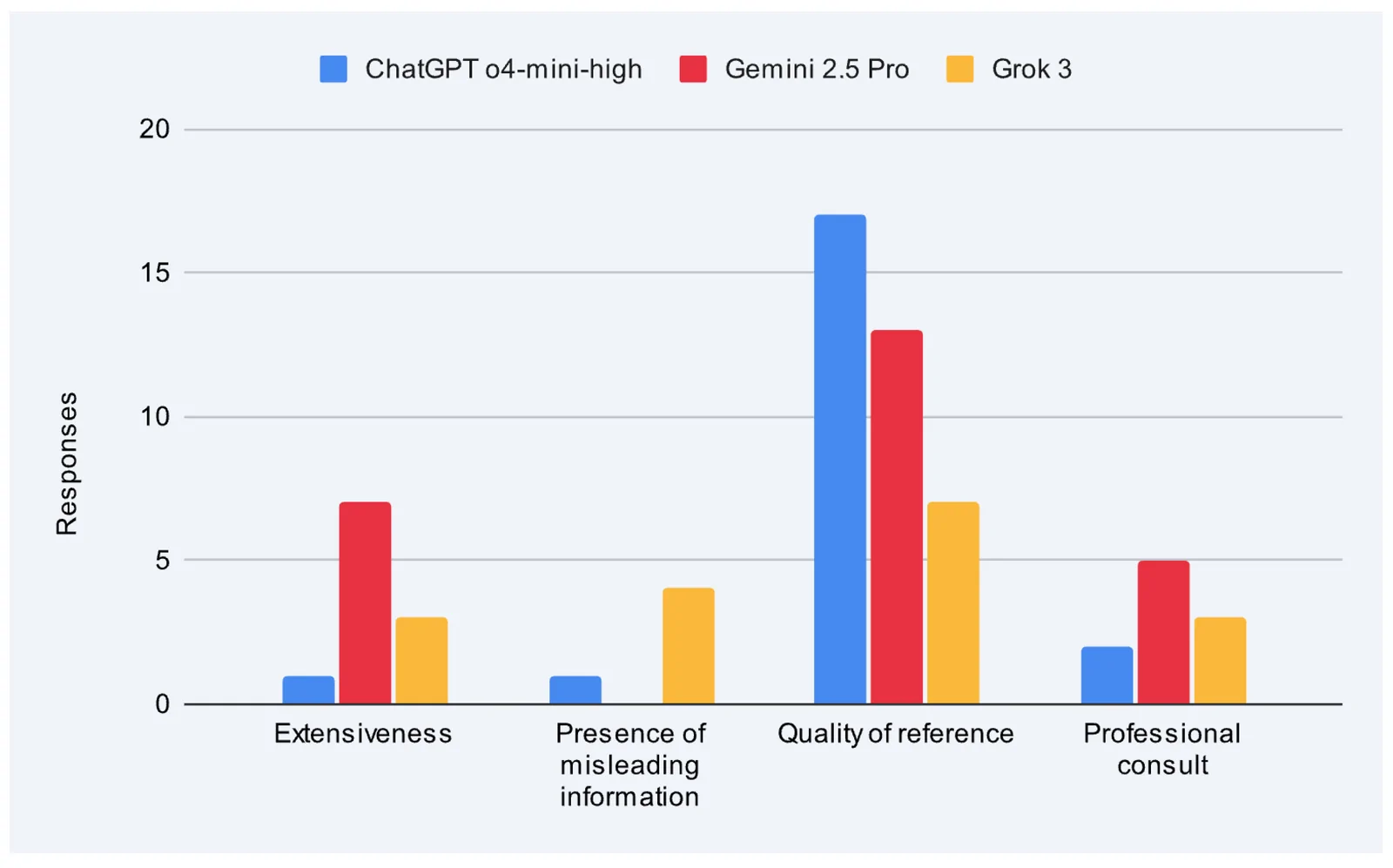
Comparative analysis of extensiveness, presence of misleading information, quality of reference, and recommendation for professional consultation among the three models.

**Figure 4:**
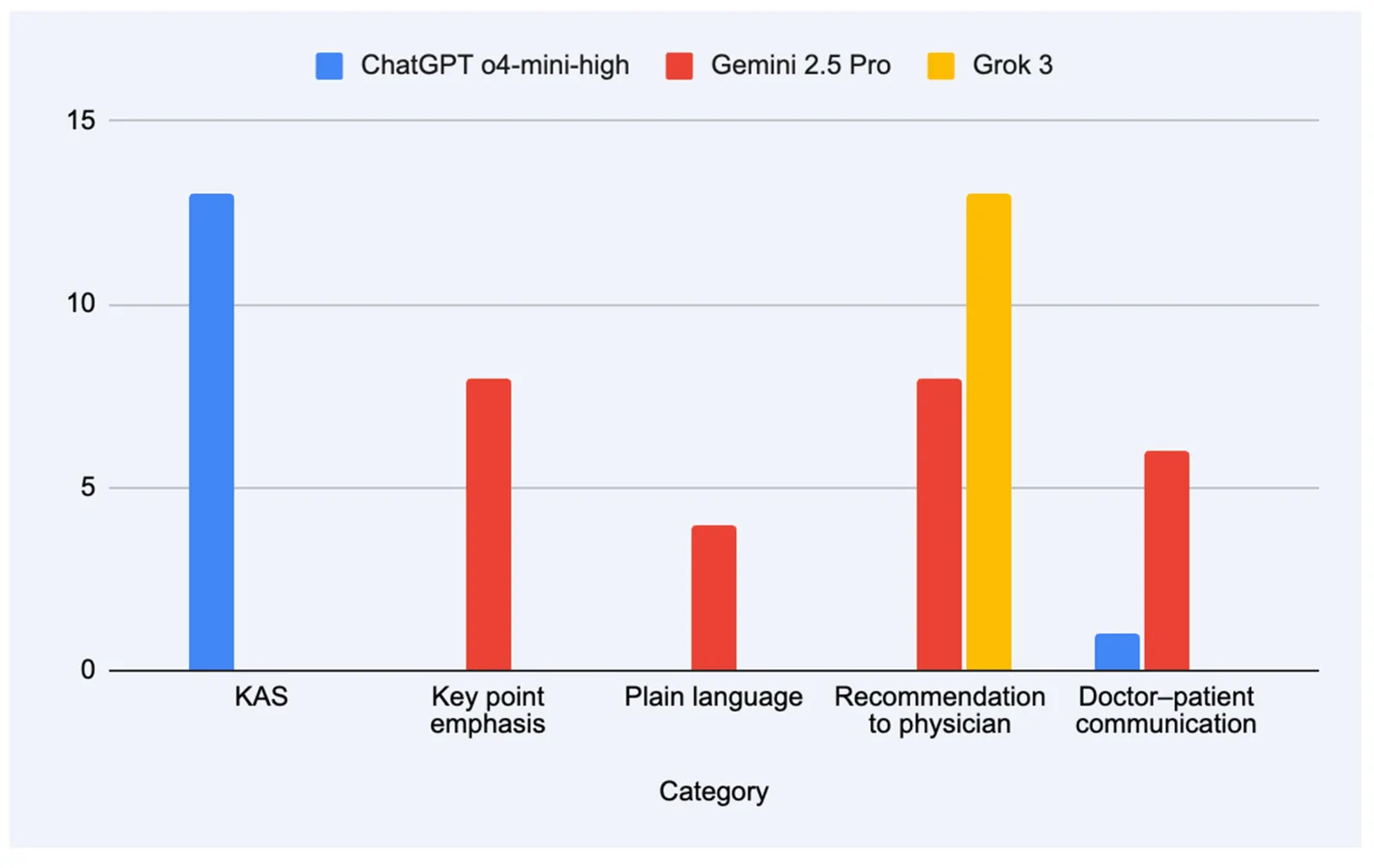
Comparative analysis of quoting KAS, key point emphasis, plain-language analogies, recommendations to physician and doctor–patient communication among the three models.

Overall, Gemini 2.5 Pro most frequently produced extensive responses (41.2%), emphasized key points (47.1%), and addressed doctor–patient communication (35.3%). ChatGPT o4-mini-high directly quoted the AAO-HNS guideline in 76.5% of responses. Grok 3 most frequently provided additional recommendations to physicians (76.5%), but also had the highest proportion of misleading information (23.5%) and the lowest proportion of reliable citations (41.2%). A comprehensive comparison is presented in Table 4 and Figure 3 and Figure 4.

### 7.3. Lexical Accessibility

The proportion of difficult words differed significantly among the three models (Friedman χ^2^ = 11.41, *p* = 0.0033, Kendall’s W = 0.34). The median proportion was 40.0% (IQR, 37.6–43.6%) for ChatGPT o4-mini-high, 36.4% (35.1–38.0%) for Gemini 2.5 Pro, and 42.1% (41.5–43.1%) for Grok 3. Gemini 2.5 Pro had a lower proportion of difficult words than Grok 3 (adjusted *p* = 0.0002) and ChatGPT o4-mini-high (adjusted *p* = 0.040), whereas no significant difference was observed between ChatGPT o4-mini-high and Grok 3 (adjusted *p* = 0.089). Overall, Gemini 2.5 Pro produced the most lexically accessible responses, although its difference from ChatGPT o4-mini-high was modest.

Response length also differed significantly among the models (Friedman χ^2^ = 27.88, *p* < 0.0001, Kendall’s W = 0.82). The median word count was 121 (IQR, 66–156) for ChatGPT o4-mini-high, 633 (567–722) for Gemini 2.5 Pro, and 898 (696–1115) for Grok 3. All pairwise comparisons were significant (ChatGPT o4-mini-high vs. Gemini 2.5 Pro and Grok 3, both adjusted *p* < 0.0001; Gemini 2.5 Pro vs. Grok 3, adjusted *p* = 0.011). A detailed comparison is provided in Table 5.

## 8. Discussion

### 8.1. Principal Findings

This study evaluated three widely used GenAI systems across three complementary dimensions: guideline concordance, clinical content and communication characteristics, and lexical accessibility. Under the tested conditions, all three systems showed high concordance with the AAO-HNS CPG for MD, with 50 of 51 responses correctly reflecting the corresponding guideline recommendations. Despite similar concordance, significant differences were observed in six of the nine clinical content and communication characteristics, as well as in response length and the proportion of difficult words. These findings indicate that guideline concordance, communication characteristics, source quality, and lexical accessibility represent distinct aspects of generated medical information and should not be considered interchangeable. In other words, the results support a multidimensional approach to evaluating guideline-based GenAI responses, as high concordance alone does not ensure verifiable references, appropriate communication, or linguistically accessible information.

### 8.2. Interpretation of Guideline Concordance

The high concordance observed across the three systems should be interpreted in the context of the question design. Correct responses primarily reflected the systems’ ability to retrieve and reproduce established guideline recommendations under direct questioning, not their ability to perform multi-step clinical reasoning. Concordance rates should also be examined alongside the nature and potential clinical relevance of individual errors. The only guideline-discordant response, generated by Grok 3, suggested that positive pressure therapy could be considered after failure of conservative treatment, contrary to KAS 10, which recommends against its use. Importantly, the response presented this recommendation with detailed explanations and references, including attribution to the AAO-HNS guideline itself. This example demonstrates that a response may appear detailed and well supported while conveying a recommendation that conflicts with the reference guideline. Although only one discordant response was identified, the potential clinical importance of an error is not necessarily proportional to its frequency within a small question set. Evaluation of medical GenAI responses should therefore consider both the frequency and nature of guideline-discordant information.

The inter-rater agreement results also require consideration of the highly imbalanced distribution of concordance ratings. The two raters agreed on 50 of 51 responses (98.0%), yet Cohen’s κ was 0 because nearly all responses were classified as correct and one rater assigned the correct category to all responses before consensus. Under this distribution, expected agreement approached the observed agreement, substantially reducing κ despite the high percentage agreement [34]. PABAK was therefore reported alongside Cohen’s κ and showed high agreement (0.96) [33]. Together with the rater-level classifications provided in Supplement S3, these measures provide a more complete description of pre-consensus agreement than either statistic alone.

### 8.3. Communication and Lexical Findings

The responses of the three GenAI systems differed in several clinical content and communication characteristics. ChatGPT o4-mini-high responses were the shortest, most frequently quoted the KAS, and most consistently provided verifiable references. Gemini 2.5 Pro responses more frequently emphasized key points and doctor–patient communication, whereas Grok 3 responses more frequently provided recommendations beyond the guideline and had the lowest proportion of reliable citations. These findings suggest that responses with similar guideline concordance can differ substantially in how clinical information is presented and supported. Therefore, concordance alone may not adequately characterize the quality of guideline-based GenAI responses.

Reference quality is particularly relevant when GenAI systems are used to communicate medical information. The single guideline-discordant response illustrates this concern: although the response provided a detailed explanation and cited several references, it incorrectly attributed a recommendation for positive pressure therapy to the AAO-HNS guideline. This example demonstrates that the presence of references does not necessarily indicate that they are accurate or support the claims being made [14,18,22]. Citation availability, bibliographic accuracy, source credibility, and consistency between the cited source and the corresponding claim should therefore be considered separately when evaluating GenAI-generated medical information.

Differences were also observed in the linguistic characteristics of the responses. Gemini 2.5 Pro had a lower proportion of difficult words than ChatGPT o4-mini-high and Grok 3, while response length differed substantially among all three systems. The CRIE was used to quantify lexical characteristics, not a validated medical reading level, and difficult words accounted for approximately 36–42% of the outputs across the three systems. These results describe differences in lexical accessibility under the present testing conditions, and do not demonstrate that one system is generally more understandable to patients.

The mechanisms underlying these differences cannot be determined from the present study. The evaluated systems are proprietary products, and their outputs may be influenced by underlying models, system instructions, retrieval configurations, and other platform-level components [13,35]. Controlled experiments would be required to determine how these components contribute to the observed differences.

### 8.4. Relation to Previous Research

Previous studies have evaluated GenAI systems using medical examinations, specialist assessments, and established clinical guidelines [15,16,17,18,19,20,21,22,23,24]. Collectively, these studies demonstrate that GenAI systems can reproduce substantial amounts of established medical knowledge, although their performance varies according to the system, clinical domain, language, and task design. The present findings extend this literature in two respects. First, the systems were evaluated using Traditional Chinese questions derived directly from an established clinical guideline. This is relevant since previous studies have suggested that GenAI performance may vary across languages [10,11,12,19,24], while the previous evaluation of GenAI responses regarding MD principally examined English-language responses and one platform [18]. Second, the present study evaluates guideline concordance, clinical content and communication characteristics, and lexical accessibility. However, direct comparison of performance across studies should be made cautiously because the evaluated tasks differ substantially. Examination questions assess factual knowledge and problem solving, whereas the present questions closely restated guideline recommendations and primarily assessed retrieval and communication of established guidance [14,15,16,25]. Therefore, our findings support task-specific evaluation, which may be an important direction for future studies comparing GenAI performance. These findings also support the need for expert oversight when GenAI systems are used to process or deliver guideline-based medical information.

### 8.5. Generalizability and Transferability

The generalizability of the empirical findings may be limited. The results were obtained from three consumer GenAI systems, one clinical condition, one guideline, Traditional Chinese prompts, and one response per question under the system configurations available on 12 April 2025. In addition, web search was enabled for all systems. The observed results therefore represent the responses generated under these specific experimental conditions and do not establish stable characteristics or overall rankings of the systems. Model updates, repeated sampling, different prompting strategies, retrieval settings, languages, or clinical topics may produce different results [15,20,35].

In contrast, components of the evaluation approach may be transferable to other guideline-based clinical domains. The core process of deriving questions from explicit guideline recommendations, preserving their clinical intent and polarity, comparing responses against a common reference standard, and evaluating guideline concordance separately from communication and linguistic characteristics is not inherently specific to MD. This approach could therefore be applied to other clinical guidelines containing clearly defined recommendations. The reference standard must be appropriate for the clinical domain and target population, while communication criteria may require adaptation to the intended users and clinical context. Linguistic measures are also language-dependent and should be replaced or modified when evaluating other languages. Furthermore, guidelines containing conditional recommendations, conflicting evidence, or complex patient-specific decision pathways may require evaluation methods beyond the direct KAS-derived questions used in this study. Future studies across different specialties, guidelines, and languages are therefore needed to determine the broader applicability and reproducibility of this multidimensional evaluation approach.

### 8.6. Limitations

There are several limitations in this study. First, the study focused only on MD-related questions, so the findings may not apply to other medical fields. Next, the questions were derived verbatim from the KASs, allowing direct comparison with the guideline but making the task similar to guideline retrieval. Also, we evaluated three models from the widely used GenAI systems in the study. The performance of other models, such as medically fine-tuned models, open-weight systems and China-developed platforms, remains unknown. Moreover, as we used only one Chinese-language variant (Traditional Chinese), the performance of GenAI across different language variants remains uncertain. In addition, we used single-query sampling to reflect real-world user behavior, so each model was represented by one response per question. Inter-rater agreement was assessed for guideline concordance but not for the nine communication characteristics, for which only the final consensus ratings were retained. Finally, there is still no standard for Chinese medical readability in the scientific community, causing difficulty for researchers in interpreting reading-level metrics for lay audiences [11,29,36].

### 8.7. Open Research Questions and Future Directions

Several questions remain for future research. First, the stability of GenAI responses should be evaluated using repeated queries and longitudinal testing across model updates [35]. Such studies could determine whether the response characteristics observed in a single-query design remain consistent over repeated generations and over time. Furthermore, future evaluations should extend beyond direct guideline-derived questions to include multi-step clinical vignettes, ambiguous presentations, differential diagnosis, and patient-specific decision-making. This would help determine whether high concordance in guideline retrieval is associated with performance in more complex clinical reasoning tasks.

The transferability of the evaluation approach also requires further investigation. Applying the same multidimensional structure to other clinical specialties, guidelines, and languages could determine which components remain applicable and which require domain- or language-specific adaptation. In particular, comparisons between Traditional and Simplified Chinese and across other non-English languages may clarify the influence of language variation on guideline concordance, communication characteristics, and lexical accessibility. The effect of retrieval should also be examined by comparing systems under controlled web-enabled and non-web-enabled conditions, where available, to distinguish the contribution of external information retrieval from that of the underlying language-generation capability.

Further work is also needed to improve the evaluation of communication and accessibility. The clinical content and communication characteristics used in this study should be validated across larger and more diverse response sets, while the development of a validated readability measure for Chinese medical text would allow lexical characteristics to be related more directly to reading difficulty. Ultimately, studies involving clinicians and patients are needed to determine whether differences in guideline concordance, source verifiability, communication, and lexical accessibility translate into differences in clinical usefulness, patient comprehension, or decision-making.

## 9. Conclusions

Under the tested conditions, three widely used GenAI systems showed high concordance with the AAO-HNS CPG for MD when responding in Traditional Chinese. However, their responses differed in source verifiability, communication characteristics, response length, and lexical difficulty. These findings indicate that guideline concordance alone does not fully characterize the quality of generated medical information, and should be considered alongside the nature of individual errors and other response characteristics. The results are specific to the present experimental conditions, and the transferability of the multidimensional evaluation approach to other clinical domains and languages requires further validation.

## Figures and Tables

**Table 1:** The English versions of prompt questions tailored to the KASs in the AAO-HNS CPG for MD.

Number	Prompt Questions Tailored to Key Action Statements
1	What are the diagnostic criteria of definite and probable Ménière’s disease?
2	Should clinicians determine if patients meet diagnostic criteria for vestibular migraine when assessing for Ménière’s disease?
3	Should clinicians carry out audiometric tests for all patients with a suspected clinical diagnosis of MD?
4	Can clinicians offer magnetic resonance imaging (MRI) in patients with possible Ménière’s disease and audiometrically verified asymmetric sensorineural hearing loss?
5	Should clinicians routinely arrange vestibular function testing or electrocochleography for the diagnosis of Ménière’s disease?
6	Should clinicians educate patients with Ménière’s disease about the natural history, measures for symptom control, treatment options, and outcomes?
7	Should clinicians offer vestibular suppressants to patients with Ménière’s disease for management of vertigo only during Ménière’s disease attacks?
8	Should clinicians educate patients with Ménière’s disease on dietary and lifestyle modifications that may reduce or prevent symptoms?
9	What medication can clinicians offer as maintenance therapy to reduce symptoms or prevent Ménière’s disease attacks?
10	Should clinicians prescribe positive pressure therapy for patients with Ménière’s disease?
11	Should clinicians offer intratympanic steroids to patients with active Ménière’s disease not responsive to noninvasive treatment?
12	Should clinicians offer intratympanic gentamicin to patients with active Ménière’s disease not responsive to nonablative therapy?
13	Can clinicians offer labyrinthectomy in patients with active Ménière’s disease who have failed less definitive therapy and have nonusable hearing?
14	Should clinicians offer vestibular rehabilitation/physical therapy for Ménière’s disease patients with chronic imbalance?
15	Is vestibular rehabilitation/physical therapy recommended for managing acute vertigo attacks in patients with Ménière’s disease?
16	Should clinicians counsel patients with Ménière’s disease and hearing loss on the use of amplification and hearing assistive technology?
17	Should clinicians document resolution, improvement, or worsening of vertigo, tinnitus, and hearing loss and any change in quality of life in patients with Ménière’s disease after treatment?

**Table 2:** Characteristics identified in GenAI-generated responses. Details in operational criteria and examples are listed in Supplement S2.

Number	Characteristics Identified
1	Extensiveness: The generated response encompassed a wider range of topics than both the KAS and its supporting texts.
2	Presence of Misleading Information: The response contains at least one clinical claim that contradicts the KAS or its supporting text, or that cannot be verified against the supporting medical literature but is presented as established information.
3	Quality of Reference: The response provides at least one identifiable citation, and every cited source is verifiable, accurately identified, credible, and supports the claim for which it is cited. Responses without identifiable citations did not meet this criterion. Adapted from the Journal of the American Medical Association (JAMA) benchmarks [32].
4	Consulting Professionals: The response includes recommendations to consult or refer to professionals for confirmation of information.
5	Quoting KAS: The response directly quotes the KAS.
6	Key Point Emphasis: The response emphasizes or reiterates the major key points of the generated content. This is adapted from the “main message and call to action” in the Clear Communication Index established by the Centers for Disease Control and Prevention (CDC-CCI).
7	Plain-Language Analogies: The response contains simple and understandable analogies to explain complex or difficult medical terms. This is similar to the item “word choice and style” in the Patient Education Materials Assessment Tool (PEMAT) [31].
8	Recommendations to Physicians: The response includes recommendations to physicians other than those mentioned in the KAS and its supporting texts.
9	Doctor–Patient Communication: The response explicitly addresses two-way communication between clinicians and patients or patient participation in decision-making, such as shared decision-making or joint treatment planning. Providing information to the patient alone does not meet this criterion.

**Table 3:** An example showing the details of Grok 3 failing one question based on KAS 10.

Proposed Question	English Translation of the Grok’s Response	Response Assessment
For patients with Ménière’s disease, should physicians arrange positive pressure therapy? (KAS 10: Clinicians should not prescribe positive pressure therapy for patients with Ménière’s disease.)	Whether **positive pressure therapy** (such as the Meniett device) should be prescribed for patients with Ménière’s disease requires individualized assessment based on symptom severity, response to conservative treatment, and clinical guidelines. A detailed analysis and the supporting basis follow, addressing whether clinicians should prescribe positive pressure therapy: ### **What is positive pressure therapy?** - Positive pressure therapy is a non-invasive treatment that delivers intermittent positive pressure pulses to the middle ear through a dedicated device (such as the **Meniett device**), aiming to improve the pressure balance of endolymphatic hydrops in the inner ear and reduce vertigo attacks. - The patient requires a tympanostomy tube, or uses the device themselves, to transmit positive pressure to the middle ear and thereby indirectly influence inner-ear fluid dynamics. ### **Should positive pressure therapy be prescribed?** According to the **AAO-HNS (2020)** and other authoritative guidelines, positive pressure therapy is **not a first-line treatment for Ménière’s disease**, but in specific circumstances it may be an option after conservative treatment has failed. Detailed considerations follow: #### **Indications and suitable situations** 1. **Suitable patients**: - **Refractory vertigo**: patients whose vertigo attacks remain frequent (≥1 per month) or seriously affect quality of life despite a low-salt diet, diuretics (such as hydrochlorothiazide), or betahistine. - **Unilateral Ménière’s disease**: positive pressure therapy is usually suitable for unilateral disease, since pressure is applied through the tympanic membrane on the affected side. - **Avoiding invasive treatment**: patients who are unwilling to accept, or unsuitable for, intratympanic injection (such as gentamicin or corticosteroids) or surgery. 2. **Clinical conditions**: - A confirmed diagnosis of **definite Ménière’s disease** is required (meeting the Bárány Society/AAO-HNS criteria: recurrent vertigo, fluctuating low-frequency sensorineural hearing loss, tinnitus or aural fullness). - After excluding other causes (such as acoustic neuroma), usually requiring MRI confirmation. - The patient needs tympanostomy tube insertion (a minor, low-risk procedure) to allow the positive pressure to be transmitted. #### **Efficacy** - **Research evidence**: - Clinical trials show that about 50–60% of patients using the Meniett device report reduced frequency or severity of vertigo attacks, especially in the early treatment period (3–6 months). - Benefit for tinnitus or hearing loss is limited; some patients may feel their aural fullness lessen. - Long-term effects (>1 year) are uncertain; in some patients the effect may weaken because of tolerance or disease progression. - **Limitations**: - Efficacy varies between individuals; some patients show no significant improvement. - Large randomized controlled trials (RCTs) are lacking, and the quality of evidence is moderate. - The patient must operate the device daily (about 5 min each time, 3–4 times a day), and adherence may affect the result. #### **Risks and side effects** - **Side effects**: usually mild, including middle-ear discomfort, complications related to the tympanostomy tube (such as infection or tympanic membrane perforation), or transient vertigo caused by improper use of the device. - **Contraindications**: middle-ear infection, unhealed tympanic membrane perforation, or inability to cooperate with device use. #### **Comparison with other treatments** - **Conservative treatment** (low-salt diet, diuretics, betahistine): should be the first-line choice, as it is low-risk, low-cost, and effective for most patients. - **Intratympanic injection** (corticosteroids or gentamicin): more invasive than positive pressure therapy, but with higher vertigo control rates (corticosteroids: 50–70%; gentamicin: 80–90%). - **Surgery** (such as endolymphatic sac decompression or vestibular nerve section): used only in refractory cases; more effective but with greater risk. - **Where positive pressure therapy fits**: as a transitional option for patients in whom conservative treatment has failed but who do not yet need invasive treatment. ### **Clinical guideline recommendations** 1. **AAO-HNS (2020)**: - Positive pressure therapy (such as the Meniett device) is listed as an **optional treatment**, suitable for patients who do not respond to conservative treatment (low-salt diet, diuretics). - Not recommended as first-line treatment, because the strength of evidence is lower than for diuretics and tympanostomy tube insertion adds risk. - Recommended that the patient participate in the decision after being fully informed of the efficacy and limitations. 2. **Bárány Society (2015)**: - Does not explicitly recommend positive pressure therapy, but acknowledges its adjunctive role in selected cases, particularly refractory vertigo. 3. **Other research**: - Studies by Thomsen et al. (2005) and Gates et al. (2006) show the Meniett device is effective for some patients, although placebo effects may influence interpretation of the results. ### **Practical clinical recommendations** 1. **Should not be prescribed routinely**: - Positive pressure therapy should not be the initial treatment for Ménière’s disease; a low-salt diet (daily sodium 1.5–2 g), diuretics (such as hydrochlorothiazide), or betahistine (8–48 mg/day) should be tried first. - Consider it only when conservative treatment has failed (no effect after 3–6 months) and the patient declines invasive treatment. 2. **Patient selection and education**: - Suitable for patients who participate actively in treatment and can cooperate with tympanostomy tube insertion and daily device use. - Physicians should explain to patients: - Efficacy (about 50–60% improvement in vertigo, with no effect on hearing loss). - The need for continued use (months to years) and adherence requirements. - Potential risks (such as middle-ear complications) and cost (the Meniett device is relatively expensive). 3. **Follow-up and assessment**: - After starting positive pressure therapy, assessment of vertigo frequency, symptom diary, and hearing changes every 3–6 months is recommended. - If there is no significant improvement, advanced treatment (such as intratympanic injection or surgery) should be considered. ### **References and guidelines** 1. **Basura GJ, et al. (2020). Clinical Practice Guideline: Ménière’s Disease. *Otolaryngology–Head and Neck Surgery*, 162(2_suppl), S1-S55.** - Lists positive pressure therapy as an optional treatment for refractory vertigo, emphasizing that it should be considered only after conservative treatment has failed. 2. **Lopez-Escamez JA, et al. (2015). Diagnostic criteria for Ménière’s disease. *Journal of Vestibular Research*, 25(1), 1–7.** - Does not explicitly recommend positive pressure therapy, but supports individualized treatment after differential diagnosis. 3. **Thomsen J, et al. (2005). Local overpressure treatment reduces vestibular symptoms in patients with Ménière’s disease. *Otology & Neurotology*, 26(1), 68–73.** - Shows the Meniett device is effective for vertigo control, but requires further validation. 4. **Gates GA, et al. (2006). The effects of transtympanic micropressure treatment in Ménière’s disease. *Laryngoscope*, 116(9), 1500–1509.** - Supports the efficacy of positive pressure therapy in some patients, although effects vary between individuals. ### **Conclusion** Clinicians **should not routinely prescribe positive pressure therapy** as initial treatment for Ménière’s disease; it should be considered as a transitional option when the patient has not responded to conservative treatment (such as a low-salt diet and diuretics) and is unwilling to accept invasive treatment. Physicians should fully explain the efficacy (about 50–60% improvement in vertigo), the risks (such as tympanostomy tube complications), and adherence requirements, and assess the effect using a symptom diary and follow-up. If you have other questions (such as the practical details of positive pressure therapy or comparison with other treatments), please let me know!	Evaluation of Grok 3’s answer: **Incorrect**, not consistent with the guideline Extensiveness: No presence of misleading information: Yes (no significant difference between the Meniett device and placebo) Quality of references: No (the 4th reference is a hallucinated one.) Professional consult: No Quoting KAS: No Key point emphasis: No Plain-language analogies: No Recommendation to physicians: Yes Doctor–patient communication: No Explanation: The Grok 3 response to KAS 10 demonstrated different types of reference-quality errors. One cited study contained inaccurate bibliographic information, while the AAO-HNS guideline was cited to support a recommendation that it does not make regarding the use of positive pressure therapy after failure of conservative treatment. The response was therefore rated “No” for quality of reference, “Yes” for presence of misleading information, and “Incorrect” for guideline concordance.

**Table 4:** Guideline concordance and clinical content and communication characteristics of the responses, by GenAI systems (*n* = 17 questions per system).

Characteristic	ChatGPT o4-Mini-High, *n* (%)	Gemini 2.5 Pro, *n* (%)	Grok 3, *n* (%)	Cochran’s Q	*p* (Unadjusted)	*p* (Holm-Adjusted)	Kendall’s W
Guideline concordance (correct responses)	17 (100.0)	17 (100.0)	16 (94.1)	2	0.3679	0.3679	0.06
**Clinical content and communication characteristics**							
Quoting KAS	13 (76.5)	0 (0.0)	0 (0.0)	26	<0.0001	<0.0001	0.76
Recommendations to physicians	0 (0.0)	8 (47.1)	13 (76.5)	18.43	<0.0001	0.0008	0.54
Key point emphasis	0 (0.0)	8 (47.1)	0 (0.0)	16	0.0003	0.0023	0.47
Quality of reference	17 (100.0)	13 (76.5)	7 (41.2)	12.67	0.0018	0.0107	0.37
Extensiveness	1 (5.9)	7 (41.2)	3 (17.6)	9.33	0.0094	0.0470	0.27
Doctor–patient communication	1 (5.9)	6 (35.3)	0 (0.0)	8.86	0.0119	0.0477	0.26
Plain-language analogies	0 (0.0)	4 (23.5)	0 (0.0)	8	0.0183	0.0549	0.24
Presence of misleading information	1 (5.9)	0 (0.0)	4 (23.5)	6.5	0.0388	0.0775	0.19
Consulting professionals	2 (11.8)	5 (29.4)	3 (17.6)	2.33	0.3114	0.3114	0.07

**Table 5:** Chinese lexical characteristics assessment.

	ChatGPT o4-Mini-High	Gemini 2.5 Pro	Grok 3
All responses			
Chinese character count	3431	20,035	26,717
Total word count	1965	11,811	15,453
Difficult word count	805	4244	6529
Difficult word percentage	41.0%	35.9%	42.3%
Sentence count	185	693	1325
Avg word count per sentence	10.6	17.0	11.7
Per response			
Word count, median (IQR)	121 (66–156)	633 (567–722)	898 (696–1115)
Difficult word count, median (IQR)	52 (25–62)	245 (223–264)	374 (299–460)
Difficult word percentage, median (IQR)	40.0 (37.6–43.6)	36.4 (35.1–38.0)	42.1 (41.5–43.1)
Difficult word percentage, mean ± SD	39.6 ± 4.7	36.0 ± 5.9	42.0 ± 2.4

## Data Availability

Data are available upon reasonable request to the corresponding author.

## Supplementary Materials

The following supporting information can be downloaded at https://www.mdpi.com/article/10.3390/life16101649/s1. Supplement S1: The questions queried and the responses of the GenAI models regarding the KASs for MD. Supplement S2: Operational scoring criteria and representative examples for the clinical content and communication characteristics. Supplement S3: Response-level data and rater-level classifications. Supplement S4: Pairwise comparisons of clinical content and communication characteristics among the three GenAI systems.
