# Supplementary material for: Multidimensional Evaluation of Guideline-Based Generative Artificial Intelligence Responses in Traditional Chinese: A Ménière’s Disease Study
Source: Life (Basel). 2026 Sep 30;16(10):1649. doi: 10.3390/life16101649 (PMC13706137; doi:10.3390/life16101649)
Supplement: Supplementary file 1 [file life-16-01649-s001.zip › Supplement S2.pdf]

| Criteria | Description |
| --- | --- |
| Authorship | Authors and contributors, their affiliations, and relevant credentials should be provided |
| Attribution | References and sources for all content should be listed clearly, and all relevant copyright information noted |
| Disclosure | Web site "ownership" should be prominently and fully disclosed, as should any sponsorship, advertising, underwriting, commercial funding |
| Currency | Dates that content was posted and updated should be indicated |

**S2.1.** The criteria of the JAMA benchmark.

## Clear Communication and Plain Language at CDC

The Clear Communication Index (Index) provides a set of research-based criteria to develop and assess public communication products. The Index supports the efforts of the Centers for Disease Control and Prevention (CDC) to comply with the Plain Writing Act of 2010 and achieve goals set forth in the National Action Plan to Improve Health Literacy and the CDC Action Plan to Improve Health Literacy.

The 20 items in the Index build on and expand plain language techniques described in the Federal Plain Language Guidelines. The research that supports the use of the 20 Index items is available at www.cdc.gov/ccindex.

## The Index at a Glance

### Why Was the Index Developed?

The Index was developed to:

1. Identify the most important communication characteristics that enhance clarity and aid understanding of public messages and materials.
2. Provide a research-based tool for staff to develop and assess communication products for CDC's audiences, no matter the format or distribution channel.

### Who Should Use the Index?

We designed the Index for:

- CDC staff who write, edit, design, and review communication products for the public
- Contractors who produce materials for CDC

Anyone who develops public health communication materials can use the Index.

### How Does the Index Work?

The Index contains 20 items, each with a numerical score of zero or one. The individual scores are converted to an overall score on a scale of 100. Although 100 is an ideal score, 90 or higher is passing.

The Index assesses materials in these 7 areas:

1. Main Message and Call to Action
2. Language
3. Information Design
4. State of the Science
5. Behavioral Recommendations
6. Numbers
7. Risk

**Estimated time required to complete the Index:** 15 minutes



**S2.2.** A brief introduction of CDC-CCI, including the 7 areas of assessment. The full index can be found at https://www.cdc.gov/ccindex/pdf/clear-communication-user-guide.pdf.

## Understandability

| Item # | Item | Response Options | Rating |
|---|---|---|---|
| Topic: **Content** | | | |
| 1 | The material makes its purpose completely evident. | Disagree=0, Agree=1 | |
| 2 | The material does not include information or content that distracts from its purpose. | Disagree=0, Agree=1 | |
| Topic: **Word Choice & Style** | | | |
| 3 | The material uses common, everyday language. | Disagree=0, Agree=1 | |
| 4 | Medical terms are used only to familiarize audience with the terms. When used, medical terms are defined. | Disagree=0, Agree=1 | |
| 5 | The material uses the active voice. | Disagree=0, Agree=1 | |
| Topic: **Use of Numbers** | | | |
| 6 | Numbers appearing in the material are clear and easy to understand. | Disagree=0, Agree=1, No numbers=N/A | |
| 7 | The material does not expect the user to perform calculations. | Disagree=0, Agree=1 | |

**S2.3.** Some of the items listed in PEMAT for printable materials (PEMAT-P), including the topic of word choice and style. The full content can be found at https://www.ahrq.gov/health-literacy/patient-education/pemat-p.html.

**S2.4.** Operational scoring criteria for clinical content and communication characteristics

| | Characteristic | Scored "Yes" if | Scored "No" if | Representative "Yes" example | Representative "No" example |
|---|---|---|---|---|---|
| 1 | **Extensiveness** | The response introduces at least one substantive clinical topic beyond the scope of the KAS and its supporting text. | Additional content only explains, repeats, translates, or reformats information within the KAS and supporting text. | Gemini Q10: discusses regulatory approval and availability of the Meniett device in Taiwan, which are outside the scope of KAS 10. | ChatGPT Q10: presents the recommendation and supporting evidence without introducing an additional clinical topic. |
| 2 | **Presence of misleading information** | At least one substantive clinical assertion contradicts the KAS or supporting text, or cannot be verified against the prespecified supporting medical literature but is presented as established information. | No substantive clinical assertion meeting these conditions is identified. | ChatGPT Q13: provides a specific threshold for nonusable hearing that is inconsistent with the reference used for assessment. | ChatGPT Q10: no clinical assertion was identified as conflicting with the guideline or supporting literature. |
| 3 | **Quality of reference** | The response contains at least one identifiable citation, and all cited sources are verifiable, accurately identified, credible, and support the claims for which they are cited. | At least one cited source is unverifiable, inaccurately identified, non-credible, or does not support the associated claim. Responses without identifiable in-text citations are classified as not assessable and are not scored using this criterion. | ChatGPT Q8: all cited sources are verifiable, accurately identified, and support the associated claims. | Grok Q10: includes inaccurate bibliographic information for a cited micropressure study and attributes a recommendation to the AAO-HNS guideline that the guideline does not support. |
| 4 | **Consulting professionals** | The response explicitly recommends that the user seek consultation, assessment, or referral from a healthcare professional. | Healthcare professionals are mentioned only as persons performing or providing an examination, investigation, or treatment, without advice to consult them. | Gemini Q1: recommends consultation with an otolaryngologist for clinical and audiological assessment. | ChatGPT Q1: presents the diagnostic criteria without recommending professional consultation. |
| 5 | **Quoting KAS** | The response reproduces the substantive wording of the relevant KAS verbatim and clearly presents or attributes it as a guideline statement. | The KAS is paraphrased, summarized, or only partially reproduced, even when its meaning is preserved. | ChatGPT Q8: directly reproduces the relevant KAS as a quotation. | Gemini Q1: accurately conveys the recommendation without directly quoting the KAS. |

| | | | | | |
|---|---|---|---|---|---|
| 6 | **Key point emphasis** | The response explicitly identifies or reiterates its principal recommendation or message, such as through a labelled key point, take-home message, or concluding summary. | The response presents or organizes the information without explicitly identifying or reiterating a principal message. | Gemini Q1: explicitly labels key points and reiterates the principal distinction in its summary. | ChatGPT Q16: organizes recommendations into categories without separately emphasizing a principal message. |
| 7 | **Plain-language analogies** | The response uses an analogy, metaphor, or familiar everyday comparison to explain a medical or technical concept. | The response only defines, translates, or simplifies a technical term without using an analogy or comparison. | Gemini Q7: uses an everyday analogy to explain the effect of prolonged vestibular suppressant use on central compensation. | Grok Q10: explains positive pressure therapy using technical description without an analogy or everyday comparison. |
| 8 | **Recommendations to physicians** | The response provides an additional actionable recommendation directed to clinicians that extends beyond the actions specified in the KAS and its supporting text. | The recommendation only restates the KAS/supporting text, is directed solely to the patient, or does not provide an additional clinician action. | Grok Q10: recommends reassessment of vertigo frequency, symptom diaries, and hearing at specified follow-up intervals beyond KAS 10. | ChatGPT Q10: provides no additional clinician-directed action beyond the guideline recommendation. |
| 9 | **Doctor–patient communication** | The response explicitly addresses bidirectional clinician–patient communication or patient participation in decision-making, such as shared decision-making or joint treatment planning. | The response does not address bidirectional communication; providing information or counselling to the patient alone is insufficient. | Gemini Q10: recommends communication with the physician and joint development of a treatment plan; ChatGPT Q13: explicitly frames discussion of benefits, risks, and alternatives as part of treatment decision-making. | Grok Q10: recommends that the physician explain efficacy, risks, and adherence without explicitly describing patient participation or shared decision-making. |
